# Adaptive Health Coaching Technology for Tailored Interventions

**DOI:** 10.3390/ijerph18052761

**Published:** 2021-03-09

**Authors:** Holly Jimison, Michael Shapiro, Misha Pavel

**Affiliations:** Khoury College of Computer Sciences, Northeastern University, Boston, MA 02115, USA; michaelshapiro@earthlink.net (M.S.); m.pavel@northeastern.edu (M.P.)

**Keywords:** health coaching, technology, tailored messaging

## Abstract

Recent advances in sensor and communications technology have enabled scalable methods for providing continuity of care to the home for patients with chronic conditions and older adults wanting to age in place. In this article we describe our framework for a health coaching platform with a dynamic user model that enables tailored health coaching messages. We have shown that this can improve coach efficiency without a loss of message quality. We also discovered many lessons for coaching technology, most demonstrating the need for more coach input on sample message content, perhaps even requiring that individual coaches be able to modify the message database directly. Overall, coaches felt that the structure of the automated message generation was useful in remembering what to say, easy to edit if necessary and especially helpful for training new health coaches.

## 1. Introduction

A confluence of factors has made it increasingly important to focus on new approaches to health care that directly address escalating costs associated with caring for people with chronic conditions. Most of our health care dollars are spent on chronic diseases, rehabilitation and conditions associated with aging [1]. With health care reform and new models of reimbursement, the incentives are becoming more aligned with techniques that foster self-management on the part of patients with chronic conditions [2,3]. The Institute of Medicine’s seminal Crossing the Quality Chasm report [4] focused on fostering self-management for patients. With advances in new sensor technology for monitoring activities in the home and environment and with a booming array of options for wireless communications devices, we now have the ability to provide just-in-time support for patients in achieving their health behavior goals as they manage chronic conditions and rehabilitation in the home and workplace. A promising aspect of interactive consumer health information technology is the potential to engage and support consumers in their own care by integrating their health information needs and preferences into information systems [5]. Such technologies are then able to provide targeted or tailored health information to support patients’ management of their health. We know from previous research that tailored information and interventions are more effective at improving patient outcomes, confidence and satisfaction with care [5,6,7,8]. In this article, we describe and assess a scalable, modular, adaptive health coaching platform that incorporates known principles of health behavior change, including frequent tailored feedback, encouragement, and recommendations for next steps based on a dynamic user model. The coaching interactions are tailored using continuously acquired data from wearable and home sensors and from phone and computer interactions. Our goal in this study was to test whether our new approach to providing automated tailored health coaching messages as a prompt for coaches to send to clients could serve to extend their reach to more clients, making them more efficient but without losing quality of care. 

## 2. Materials and Methods 

Our overall approach to addressing the challenges of delivering effective health coaching interventions was to focus on developing a robust and modular software platform that facilitates the management of home-based sustained interventions. This approach to developing a modular intervention delivery platform for remote home-based interventions has the benefit of being a low-cost scalable approach for delivering care to patients on a more continuous basis at home. This modular health coaching system enables a human health coach to manage a large number of clients remotely and was developed using feedback from our needs assessment with patients, caregivers and expert stakeholders [9]. In addition to the continuously monitored sensor data, the system encodes information about the individual user (health concerns, motivations, barriers, readiness-to-change, data sharing preferences, contact preferences, etc.) to develop a dynamic user model and a tailored action plan for health behavior change. We have created and tested modules for cognitive exercise [10], physical exercise [11,12,13,14,15], sleep management [16], socialization [17], and stress management [18,19].

**Figure 1:**
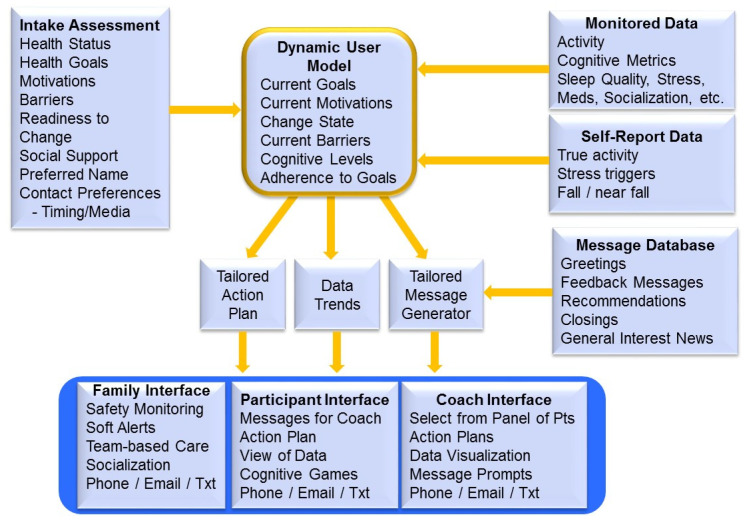
Information flow for the Health Coaching Platform illustrating the interactions among the components of the system used to generate tailored messages and interfaces. Active methods trigger just-in-time interactions based on new input from sensor and self-report data.

A high-level diagram of the general architecture for the health coaching platform is shown in Figure 1. The information flow depicted shows that the patient first goes through an initial assessment module to obtain background information on current cognitive health behaviors, optimal methods and times for contacting and interacting with the user, goal setting information, motivation to change, and readiness to change. This information, as well as data from the monitoring systems are stored in the user database to support the dynamic user model. The current version of the system incorporates an AI-with-human-in-the-loop approach where there is supervisory control from a human coach. The architecture leverages a rule-based implementation of the user model where most intervention protocols are encoded as state-dependent active methods. The incoming data from sensors and user input in the dynamic user model trigger the state-dependent active methods that generate tailored action plans and tailored coaching messages to be presented to the coach for possible editing before sending them on to the study participants in the home. The content database contains a mix of generic and possible tailored messages. The message tailoring is based on static information (e.g., literacy level, user preferences, motivations, barriers, triggers) and dynamic updates from sensors (e.g., wearables, motion sensors) and computer/phone interactions (e.g., system use adherence, cognitive metrics from adaptive computer games, user requests). The novel and innovative aspect of the message tailoring is based on the inferences made within the dynamic user model. We use known principles of health behavior change from both motivational interviewing [20] and the Transtheoretical Model of Behavior Change [21] to inform estimates of action plan adherence and readiness to change. Message content is based on whether we infer that a user has moved from precontemplative or contemplative states (where we assume background and motivational content is required) to preparation, action or maintenance phase (where estimates of adherence inform readiness–to-change and content selection). 

**Figure 2:**
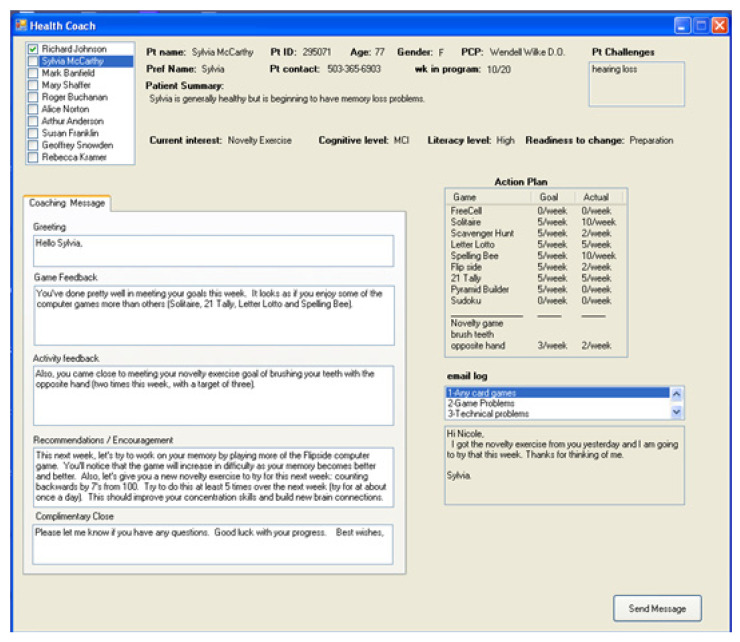
Messaging screen for the coach interface showing a client’s background information, action plan, previous emails or texts, as well as a triggered automated suggested message for the coach to send (after optional edits).

Figure 2 shows a sample screen of the Coach Dashboard, where background information on a client (in this case Sylvia McCarty) is displayed, along with the client’s action plan. In this example the client has cognitive health goals and the action plan shows targets for use of the adaptive cognitive computer games and a novelty exercise of brushing teeth with the opposite hand. 

**Figure 3:**
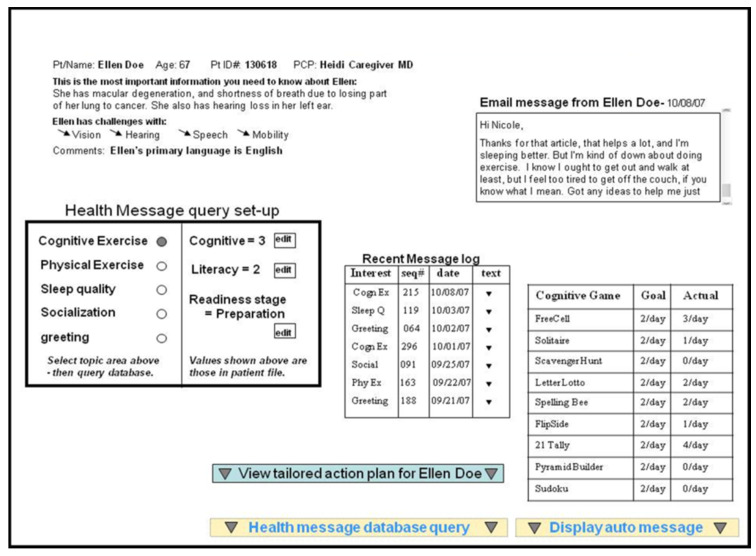
Sample patient background presented in the coach interface.

Ideally, our coaching system would generate coaching suggestions and messages that would be at least as good as those of successful human coaches. The success of our coaching approach, therefore, depends on the ability of the system to generate appropriate user-tailored messages. To reduce the effect of the variability across user population, we tested this capability by engaging experienced coaches’ interactions with simulated patients and evaluated the messages to the users. To test the overall effectiveness of our dynamic user model in generating tailored messages we created 10 simulated patients with varying experiences over a coaching period of 4 interactions and tested the efficiency and quality of coaching interactions with 6 experienced health coaches [22]. The simulated population was over the age of 64 years with a mean age of approximately 80. While all members of the simulated cohort had some level of chronic illness, all were able to function at home in conducting the typical activities of daily life. We also created the situation where they were in the midst of a coaching intervention and that they were all comfortable in using a computer for email and for seeking information on the Internet. These simulated patients were representative of our patients in an ongoing health coaching study, but we wanted to de-identify all data presented since we would later be asking these participants to evaluate coaching message quality. Additionally, we felt it was important to provide more variety of experiences among the simulated patients for the purposes of this study. Figure 2 shows a sample screen from the coaching interface where coaches had access to the background, current status and previous communications for each simulated patient. For the purposes of this study we created two versions of the coaching dashboard: (1) All the information about a patient of interest would be displayed as on this screen, and the coach would create the message by hand, and (2) The same patient information would be displayed, but the coach would be able to review and possibly edit a tailored message generated by the health coaching platform as shown in Figure 3.

The six coaches who participated in this study were familiar with managing large panels of patients and sending individual messages or communicating via phone. For this study they were first trained on the health coaching platform to the point of feeling comfortable in using it. The coaches were also primed with the following scenario: “You are responsible to provide coaching to 200 patients on a weekly basis, with a scheduled frequency of once per week. Keeping in mind that your patient load will average 40 per day, your task will be to craft a message to each patient based upon the information provided to you on the coaching screen. You will want to try to personalize your message to reflect the unique set of information presented for that individual”. With that workflow scenario in mind, we asked each coach to use our health coaching platform to create coaching messages for 10 simulated patients on 4 separate days (40 messages total for each coach), expecting that this would be a realistic workload for a coach’s day. The coaches performed this task on separate days, using the coaching platform with the automated messaging on two of the days and the coaching platform without automated message assistance on the other two days. The order of the session types was pseudo-randomized to further reduce learning effects. Additionally, the 10 simulated patients were new or at a different stage of the intervention each time. The coaching dashboard providing background information on the patients, as shown in Figure 2, was the same in both cases; it including name, gender, age, health status, health behavior goals, readiness to change, previous adherence, and previous message responses. The only difference between the two tasks on different days was the automated messaging assistance in one of the sessions, as shown in Figure 3. The automated messaging provided (1) a sample greeting (picked randomly from possible greetings in the message database with the patient’s preferred name inserted), (2) tailored feedback on the patient’s action plan performance for each health goal from the previous week based on data from the home, (3) tailored recommendations for the coming week’s action plan, and (4) a complimentary closing statement (picked randomly from the message database with the coach’s name inserted). The dynamic user model in the health coaching system is used to tailor the feedback and recommendation components of the automated message based on sensor data and patient interactions with the system. The coaches using the automated messaging system had the ability to send the message as is, edit it and send, or even not use it at all and start from scratch.

Our research questions for this study were to determine if the automated messaging availability improved the coach’s efficiency and whether there was a change in the quality of the messaging as measured by other coaches’ ratings and actual patients’ ratings. Finally, we debriefed the coaches on the system usability and their preference for manual or semi-automated messaging. 

To answer the coach efficiency question we measured the time taken to complete the set of interactions with 10 simulated patients per day. To measure the quality of the messages in both cases, we later de-identified the coach author and the version of the system that the message was created on, and then asked our 6 expert coaches to rate the quality of each message on a scale of 0–10 (unsatisfactory to excellent), given the background information and status of each simulated patient. The coaching message created by the coach doing the rating was excluded from consideration, so only 200 of the 240 total messages were rated by each coach. We additionally recruited 7 patients who had been in one of our health coaching interventions previously and were familiar with being coached to rate how satisfied they might be with the coaching message from the view of the particular simulated patient. Finally, for the debriefing of the coaching we asked specifically about the usability of the coaching platform, their preferences regarding the two approaches to messaging, as well as suggestions they had for improving the coaching experience.

## 3. Results

### 3.1. Coach Efficiency

For our test of coach efficiency, we measured overall differences in times taken to complete patient coaching interactions. Overall, the mean patient coaching time per patient with the manual session was 4:59 ± 1:45 min for the manual system and 3:14 ± 1:33 min for the semi-automated system. With a paired t-test this difference of 1:45 min was significant with a 2-sided *p* value of 0.009. 

### 3.2. Message Quality

**Figure 4:**
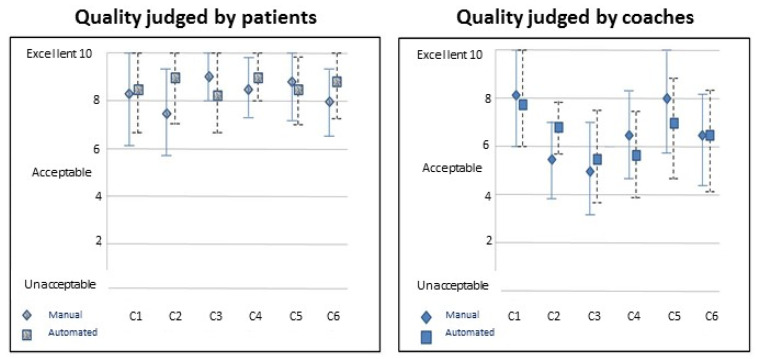
This figures shows the means and standard deviations on judgments of quality of coach messaging, ranging from Unacceptable (0) to Excellent (10). The graph on the left shows patients’ ratings of each of 6 coaches’ set of 40 messages. Diamonds represent their hand-crafted messages and the squares represent messages with the assistance of automated message generation. The graph on the right shows the ratings for the messages as judged by other coaches.

For our test of message quality from patients’ perspective, we compared patient blinded ratings for messages from each coach in each condition. The graph on the left of Figure 4 shows that patients gave all messages a high rating. All were above acceptable. However, there was no significant difference between ratings for messages with or without the automated messaging assistance. For our test of message quality from the coaches’ perspective, we compared coach ratings of blinded messages from other coaches who had not written the messages. The graph on the right of Figure 4 shows that coaches rated the messages from each coach as adequate or above, but with no significant difference, either within coach or overall.

### 3.3. Coach Debriefing Findings

The coaches in our study were very forthcoming with feedback and suggestions, both for our health coaching platform specifically, but also for scalable coaching technology in general. The lessons learned from our debriefing included the following:

- Tone: Messages should never be framed in a negative way. Focus on accomplishments not failures.
- Tailoring/Personalization: Recommendation/suggestions need to be more personalized
- Customization:
  ⚬ System would be highly useful if “the words were correct” (i.e., their own words framed in the right way)
  ⚬ Template was helpful–wanted it to be “my template”
- Training: There was consensus that especially at first, it is easier to have automated text in front of you. “When you’re learning, you’re not sure of the tone to take with people, or exactly what to say”
- Structure:
  ⚬ Having protocols built in to the automated system helped.
  ⚬ The first sections (feedback on game play and activity) could definitely be automated. (Less concern about tailoring here)
- Efficiency:
  ⚬ Prompts were “excellent time saver, reminder of what to say.”
  ⚬ “It didn’t take much time to tweak these messages”

## 4. Discussion

We found that our participating health coaches were more time efficient with the aid of the automated system, with no significant difference in perceived quality of the messages. This finding is very encouraging in making coaching for health behavior change more scalable and cost effective. Given the need for continuity of care for the important aspects of health behaviors in the management of chronic conditions, we anticipate that making these systems more cost effective will encourage further adoption and funding from health insurers and clinics. There have been rapid advances in sensor technology and battery life, along with nearly ubiquitous use of mobile phones, even within poor and disadvantaged communities. These changes all serve to make health interventions to the home a promising solution to improving the quality, cost and accessibility of healthcare. 

### Limitations

We found that the primary limitation of the study was our need for further input from coaches on the set of potential message components in our database. Given that in our debriefing of coaches we found some level of dissatisfaction with the style of the automated messages, usually having to do with not being encouraging enough when adherence was low, the experiment would likely be even more successful if we had more prior coach input on our message database. It should be noted however that the patient raters gave higher ratings than the coaches in general, and tended to rate the automated messages higher than the manual ones. Additionally, the small number of available health coaches required us to use the coach/subject as the coach/raters, which may be a confounding factor in our quality results.

Additionally, feasibility considerations determined that this would be a constrained study. We decided early on that our health coach subjects would not be utilizing motivational interviewing principles. For similar reasons the concept of readiness to change was not one of the parameters used in formulating the individual tailored messages. Additionally, the reliance on fictional patients removed a potential dimension of interaction from the coaching experience. Yet these limitations facilitated a focus which enabled insights into, from the health coach perspective, what worked and what was problematic. 

## 5. Conclusions

In this study we have demonstrated that our framework for a health coaching platform with a dynamic user model used to generate tailored health coaching messages can improve coach efficiency without a loss of message quality. We also discovered many lessons for coaching technology, most demonstrating the need for more coach input on sample message content, perhaps even requiring that individual coaches be able to modify the message database directly. Overall, coaches felt that the structure of the automated message generation was useful in remembering what to say, easy to edit if necessary and especially helpful for training new health coaches. Although our current inference algorithms for assessing patient state and tailoring just-in-time messages proved fruitful, future research is needed in developing optimal content, modeling and ensuring individual privacy preferences, and integrating adaptive learning from data into our dynamic user models.
